# Fiber Pathway Pathology, Synapse Loss and Decline of Cortical Function in Schizophrenia

**DOI:** 10.1371/journal.pone.0060518

**Published:** 2013-04-08

**Authors:** Max R. Bennett, Les Farnell, William G. Gibson

**Affiliations:** 1 The Brain and Mind Research Institute, University of Sydney, Sydney, NSW, Australia; 2 The Centre for Mathematical Biology, University of Sydney, Sydney, NSW, Australia; 3 The School of Mathematics and Statistics, University of Sydney, Sydney, NSW, Australia; University of Adelaide, Australia

## Abstract

A quantitative cortical model is developed, based on both computational and simulation approaches, which relates measured changes in cortical activity of gray matter with changes in the integrity of longitudinal fiber pathways. The model consists of modules of up to 5,000 neurons each, 80% excitatory and 20% inhibitory, with these having different degrees of synaptic connectiveness both within a module as well as between modules. It is shown that if the inter-modular synaptic connections are reduced to zero while maintaining the intra-modular synaptic connections constant, then activity in the modules is reduced by about 50%. This agrees with experimental observations in which cortical electrical activity in a region of interest, measured using the rate of oxidative glucose metabolism (CMR_glc(ox)_), is reduced by about 50% when the cortical region is isolated, either by surgical means or by transient cold block. There is also a 50% decrease in measured cortical activity following inactivation of the nucleus of Meynert and the intra-laminar nuclei of the thalamus, which arise either following appropriate lesions or in sleep. This occurs in the model if the inter-modular synaptic connections require input from these nuclei in order to function. In schizophrenia there is a 24% decrease in functional anisotropy of longitudinal fasciculi accompanied by a 7% decrease in cortical activity (CMR_glc(ox)_).The cortical model predicts this, namely for a 24% decrease in the functioning of the inter-modular connections, either through the complete loss of 24% of axons subserving the connections or due to such a decrease in the efficacy of all the inter-modular connections, there will be about a 7% decrease in the activity of the modules. This work suggests that deterioration of longitudinal fasciculi in schizophrenia explains the loss of activity in the gray matter.

## Introduction

Goldman-Rakic [1] was the first to highlight the possible dysfunction of major fiber pathways in the cortex of those suffering from schizophrenia. Such impairments in white matter have now been identified (for a meta-analysis of studies, see [2] and explained in terms of their impact on cognition [3]. Particular emphasis has been placed on the superior longitudinal fasciculus (SLF), a major fiber pathway that connects the supramarginal gyrus of the parietal lobe with the middle frontal gyrus/dorsolateral prefrontal cortex [4], [5]. The reason for this is that the posterior parietal and frontal cortices form a network [6] which supports working memory [7], [8], [9] and matures as fiber pathways between these parts of the cortex mature [10]. The parietal and frontal components of this network show pathological changes in schizophrenia, both indicated by loss of gray matter [11], [12] and white matter [13]–[18]; for a recent review, see [19]. The fronto-parietal network receives major inputs from the thalamic intralaminar nuclei and the basal nuclei whose integrity is necessary for the normal functioning of the network. [20], [21].

For over a decade functional magnetic resonance imaging (fMRI) has revealed irregularities in the activity of the fronto-parietal network in schizophrenia [22]–[25]. A number of different techniques indicate subnormal connectivity between fronto-parietal and fronto-temporal regions [24]–[28]. Consistent with these observations, diffusion tensor imaging (DTI) has revealed reduced fractional anisotropy in the SLF, the main pathway between frontal and parietal cortex, as well as in the cingulum fasciculus, the pathway subserving the fronto-temporal connection [29]. Indeed the extent of fractional anisotropy of the superior longitudinal fasciculus is correlated with decreases in working memory in schizophrenia patients, a capacity that depends on the integrity of the fronto-parietal network [19], [29]. Furthermore, disturbances in the integrity of the SLF in schizophrenia are indicated by transcranial magnetic stimulation (TMS) of the parietal-motor pathway [30].

Very extensive studies have been made of activity in the different components of the fronto-parietal network, using the rate of oxidative glucose metabolism (CMR_glc(ox)_) as a measure of the activity, based on the very close quantitative relationship between synaptic and action potential firing in a cortical area of interest and CMR_glc(ox)_ in that area [31]. Decreases in CMR_glc(ox)_ have been noted in the fronto-parietal network of patients with schizophrenia compared with controls (see, for example, [32]. The present work seeks to determine whether the observed changes in functioning of the fronto-parietal network, measured with CMR_glc(ox)_, can be explained in terms of the changes in integrity of the longitudinal fascicules subserving the network.

## Methods

**Figure 1:**
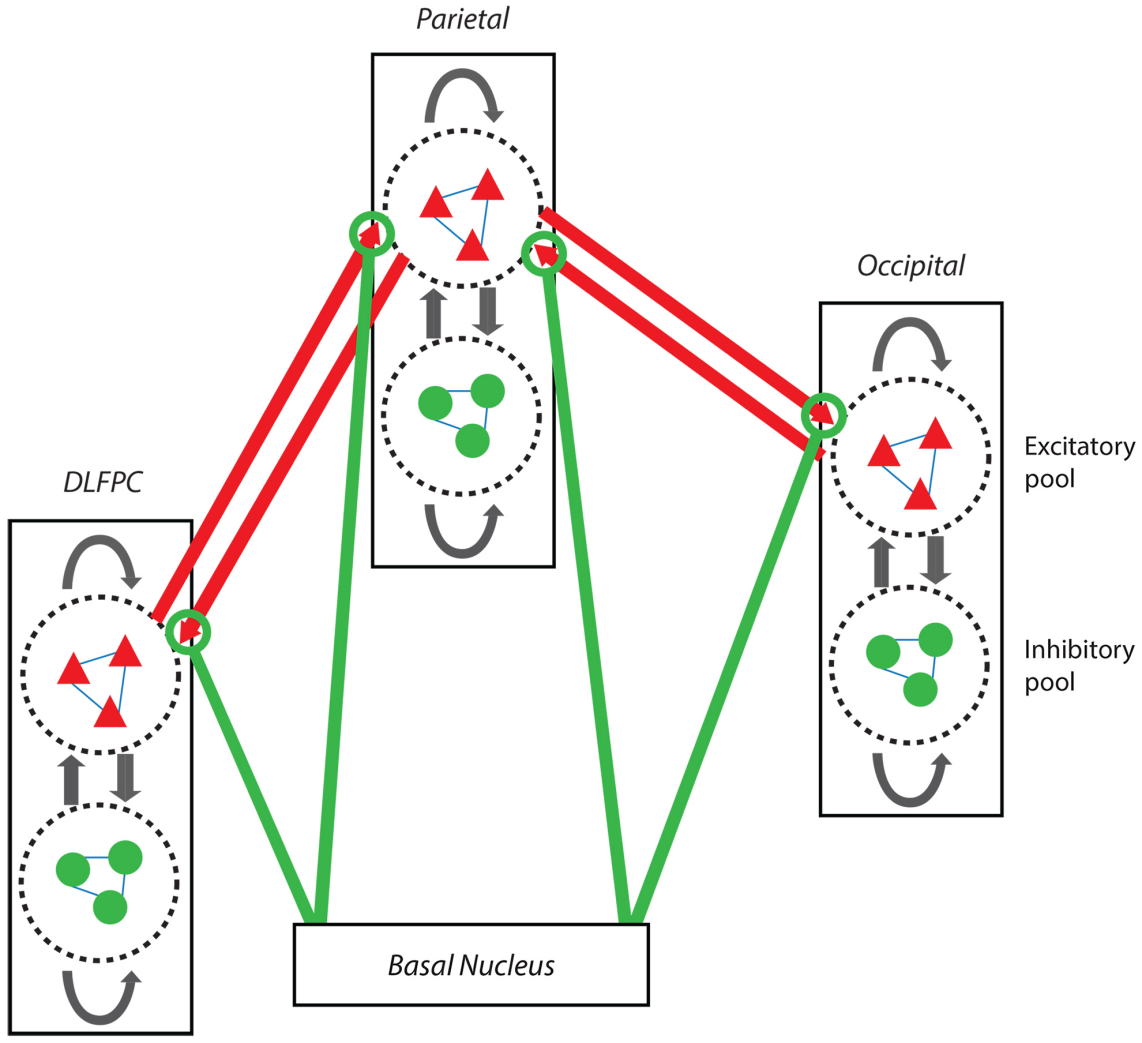
Model of a modular network such as that connecting the dorso-lateral prefrontal (DLPC)/parietal/occipital cortical modules. However, these are only given as being representative of typical cortical regions, and in the computations each module (indicated by a rectangle) contains an identical neural network with N neurons, 20% inhibitory (green), 80% excitatory (red), each connected to other neurons with connection strength *A*_k_ for excitatory neurons and *B*_k_ for inhibitory neurons. The arrows in the rectangles indicate that excitatory neurons are connected with each other and to inhibitory neurons, as well as projecting to other modules (long red arrows). Likewise, other arrows in the rectangles indicate that inhibitory neurons are connected with each other and to excitatory neurons. The lower module represents projections to the upper modules from the basal nuclei (magnocellularis in rat and Meynert in humans) and the non-specific intralaminar nuclei of the thalamus (green arrows). These are taken here to specifically change the efficacy of transmission of associational synapses in the modules (the green lines end in circles, taken to indicate their synapsing on synaptic endings between modules (red-arrow heads). This figure is for Model 2 and the mathematical theory is given under Methods.

We use two different theoretical models in order to investigate the effects of changing synaptic connectiveness in the cortical model (Figure 1). The first (Model 1) is a simple firing-rate model; however, in many cases it can provide an adequate description of network activity (see comments and references to earlier work in [33]. The second (Model 2) uses integrate-and-fire neurons; these have been extensively employed in theoretical investigations of network behaviour [34]. The principal reason for using two models was to provide some evidence that the main theoretical results reported here are not dependent on the particular neural network model employed. The models are rather different and it is not the purpose of the present research to do a detailed comparison of them, but rather to show that they give similar results in the context of the present investigation. Specifically, the models are used to compute the decrease in neuronal activity as the coupling between subsets of interconnected neurons is decreased.

### Model 1

In this model, the neurons are described by firing rates, not by action potentials. We follow the formulation of [35].

The network consists of *N* interconnected neurons, with no distinction being made between excitatory and inhibitory ones. Neuron *i* is characterized by an activation variable 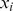 satisfying
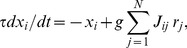
(1)where *τ* is the membrane time constant, 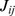 describes the connection from neuron *j* to neuron *i* and *g* is a parameter controlling the strength of the synaptic connections. The quantities 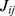 are chosen independently and randomly from a Gaussian distribution with mean 0 and variance 1/N; hence about half of the connections are excitatory and half inhibitory.

The firing rate of neuron *i* is 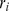 where 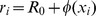 with 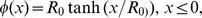 and 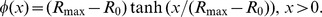 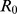 is the background firing rate and 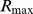 is the maximum rate. Averaging over all *r_i_, i = *1 to *N*, then gives the average activity in the network.

An important part of the present investigation concerns the interaction between modules (subsets) of neurons. Each module is capable of sustained activity without any external input. The modules are then linked by connections with synaptic strength *g_ext._* The number of connections can also be varied.

### Model 2

This model uses a network of integrate-and-fire neurons. Figure 1 depicts a system of three interconnected modules, each containing an identical neural network. (The labels DLFPC, Parietal and Occipital are to indicate representative cortical regions, but no detailed modeling of different regions is undertaken.) Each module contains pools of excitatory neurons (red triangles) and inhibitory neurons (green circles), synaptically connected within and between pools (black arrows). The long red arrows indicated connections between the excitatory neurons in different modules; the strength of these connections can be modified, for example by input from a basal nucleus, indicated by green lines. The two outer modules can also be connected (not shown in the figure).

The formalism below is a simplified version of that of [36]); see also [37].

The membrane potential *V* of a single neuron is governed by
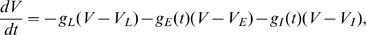
(2)where 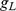 is the leak conductance, 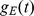 and 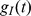 are the time-dependent conductances arising from external inputs and from the network activity of excitatory and inhibitory neurons. These have been normalized by the membrane capacitance and thus have dimensions of inverse time. 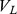 is the rest potential and 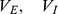 are the excitatory and inhibitory reversal potentials.

The membrane potential of a neuron evolves according to integrate-and-fire dynamics. A spike is generated when 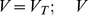 is then reset to 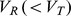 and held there for an absolute refractory period 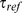. The membrane potential is normalized so that 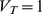
and 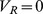.

We model a network of *N* neurons, of which 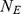 are excitatory and 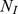 are inhibitory. The conductance change in a typical neuron due to excitatory inputs is
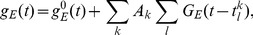
(3)where 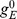 is the contribution from external excitatory sources, 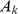 is the connection strength from the *k*th excitatory neuron and 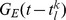 describes the effect of the post-synaptic current generated by an action potential in neuron *k* at time 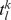. This function is taken to have an α-like form:
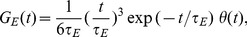
(4)where 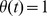
for 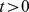
and 0 otherwise; 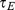 is the time constant for excitatory neurons. The sum 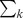 is over all neurons connected to the excitatory neuron under consideration and 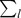
is over all firings of neuron k up to time t. Similarly, the conductance change in a typical neuron due to inhibitory inputs is
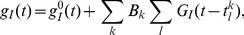
(5)where 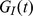 is given by Eq 3 with 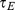 replaced by 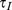. The connection strengths 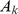 and 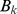 generally have a dependence on the spatial separation of the neurons (usually taken to be a Gaussian) and also can depend on the excitatory or inhibitory nature of the coupled neurons, but in our simplified model we set 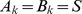, where S is a constant. (Investigations using Gaussian spatial dependence indicate that this simplification does not significantly alter the results reported here.) The sums over k in Eqs 3 and 5 are over all neural connections; in the computations, a fraction F of all N neurons are connected, with no distinction made between excitatory and inhibitory neurons.

The time-evolution of the network is initiated by a random inputs (Poisson trains) applied separately to each neuron. Temporal updating uses a modified second-order Runge-Kutta scheme [38]. The external Poisson train is applied for 500 ms, then removed and the system allowed to evolve freely for a further 500 ms, during which time it reaches a steady state in which the average firing rate remains constant; data is then collected over the next 500 ms. (Some typical firing trains are shown in Figure S1.).

Again, we investigate the interaction between modules (subsets) of neurons (Figure 1). The connections between the modules may be only excitatory or both excitatory and inhibitory as they give very similar results. The parameter *S_ext_* gives the connection strength and *F_ext_* gives the fraction of connections between neurons in different modules.

### Parameter Choice

The crucial requirement is that the network in an isolated module settles into a stable state that is neither dead (zero firings) nor one in which all neurons are firing at their maximum rate. This requirement places quite severe restrictions on the range of parameter values that can be used. For both models we did extensive tests varying the number of neurons and the strengths and number of synaptic connections. For Model 1 it was found that 1000 neurons were sufficient to give consistent results. The main other adjustable parameter is the synaptic strength *g,* and this was varied in the range 1<*g*<2.5. For larger values the firing rates became oscillatory, rather than steady. We note that [33] used *g* = 1.5 as a typical value. For Model 2 various sized networks were investigated, ranging from 900 to 10,000 neurons. In each case, a connection fraction *F* was first chosen and then the synaptic strength *S* was adjusted to obtain a stable state. For example, the choice *N = *900, *F* = 0.2 and *S* = 0.0085 gave a stable state with a firing rate of about 85 Hz. For *N = *10,000 and *F* = 0.2, *S* had to be adjusted to 0.00075 to give a similar result; for some results involving networks of different sized, see Figure S2B. (A firing rate of 85 Hz is of course much higher than experimentally observed, but this is a consequence of the necessity of using model networks with many fewer neurons than physiological ones; see, for example, [39]. Most calculations were done using networks with more than 2000 neurons, as the averaging over spike trains gave more consistent results. The number of excitatory neurons was fixed at 80% of the total for the calculations reported here, but varying this did not significantly change the results. For calculations involving 2 or 3 modules, each module is an identical network, with parameters chosen to give stable states when the modules are uncoupled. Then either the inter-modular neuronal coupling strength *S_ext_* or the inter-modular connection fraction *F_ext_* is varied. Tests were also performed where the inter-modular connections were purely excitatory, but differences were not significant. Parameter values used in the calculations are listed in Table 1.

**Table 1:** Parameter values in the Models.

Quantity	Symbol	Value	Notes
**Model 1**			
Number of neurons	*N*	1000	
Membrane time constant	*τ*	10 ms	[35]
Intra-modular synaptic strength	*g*	various	
Inter-modular synaptic strength	*g_ext_*	various	
Background firing rate	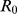	0.1	[35]
Maximum firing rate	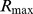	1.0	[35]
**Model 2**			
Number of excitatory neurons	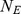	various	
Number of inhibitory neurons	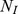	various	
Rest potential	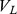	0	[37]
Excitatory reversal potential	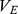	14/3	[37]
Inhibitory reversal potential	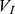	−2/3	[37]
Absolute refractory period	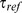	5 ms	
Leak conductance	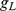	0.05 ms^−1^	[37]
Excitatory decay time	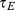	1 ms	adjusted
Inhibitory decay time	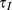	5/3 ms	adjusted
Intra-modular neuronal coupling strength	*S*	various	
Inter-modular neuronal coupling strength strength	*S_ext_*	various	
Intra-modular connection fraction	*F*	various	
Inter-modular connection fraction	*F_ext_*	various	

## Results

### Theoretical: Predicted Changes in the Functioning of Cortical Areas on Changing the Extent of their Inputs

This theoretical section is necessary to answer the following specific questions in the Results: Is about half of the activity in a particular area of cortex dependent on its associational synaptic connections with other areas of cortex? Is the observed decline in associational connections in schizophrenia sufficient to account for the observed decline in activity in the areas of cortex subserved by these connections?

#### Computational estimates (Model 1)

**Figure 2:**
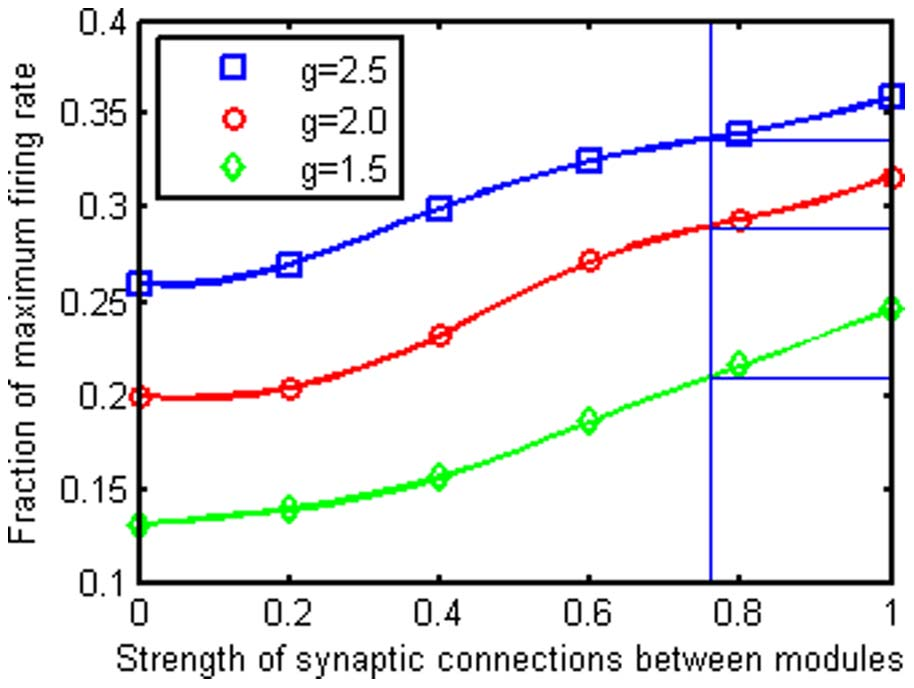
Firing rates for a two-module network. The simulation uses Model 1, with parameter values given in Table 1; each module contains 1000 neurons. The firing rate, normalized by the maximum firing rate, is plotted against the strength *g_ext_* of synaptic connections between the two modules for three values of the intra-modular synaptic strength parameter *g.* The overall decreases in firing rate as the inter-module connection decreases from fully coupled to completely uncoupled are 47%, 37% and 28% for *g* = 1.5, 2.0 and 2.5, respectively. The corresponding decreases as the connection strength decreases by 24% (vertical line) are 10%, 8.5% and 6.5%, respectively.

Figure 2 shows the computational solutions for different values of *g* (the strength of intra-modular synaptic connections) in the case of two coupled cortical modules. This indicates that for decreasing inter-modular synaptic strength *g_ext_* there is an increase in the extent of decline in activity within the modules (Figure 2). This is such that a 24% decrease in inter-modular synaptic coupling strength leads to a decrease in activity of6.5%, 8.5% and 10% for *g* values of 2.5, 2.0 and 1.5 respectively. As is shown below, if cortical activity is measured in schizophrenia it declines by similar percentages (4% to 7%) when there is a 24% decrease in the integrity of longitudinal fascicles supplying the synaptic coupling between different cortical areas, identified here as different modules. If, for *g* = 1.5, the inter-modular synaptic coupling strength is reduced to zero, then the activity within the modules is reduced by 47%, which, as shown below, is about the reduction in activity of cortical areas observed when they are deprived of any coupling. Calculations were also performed in which the number of inter-modular synaptic connections was reduced. The results differ by less than a maximum of 10% from those in Figure 2.

#### Simulation estimates (Model 2)

**Figure 3:**
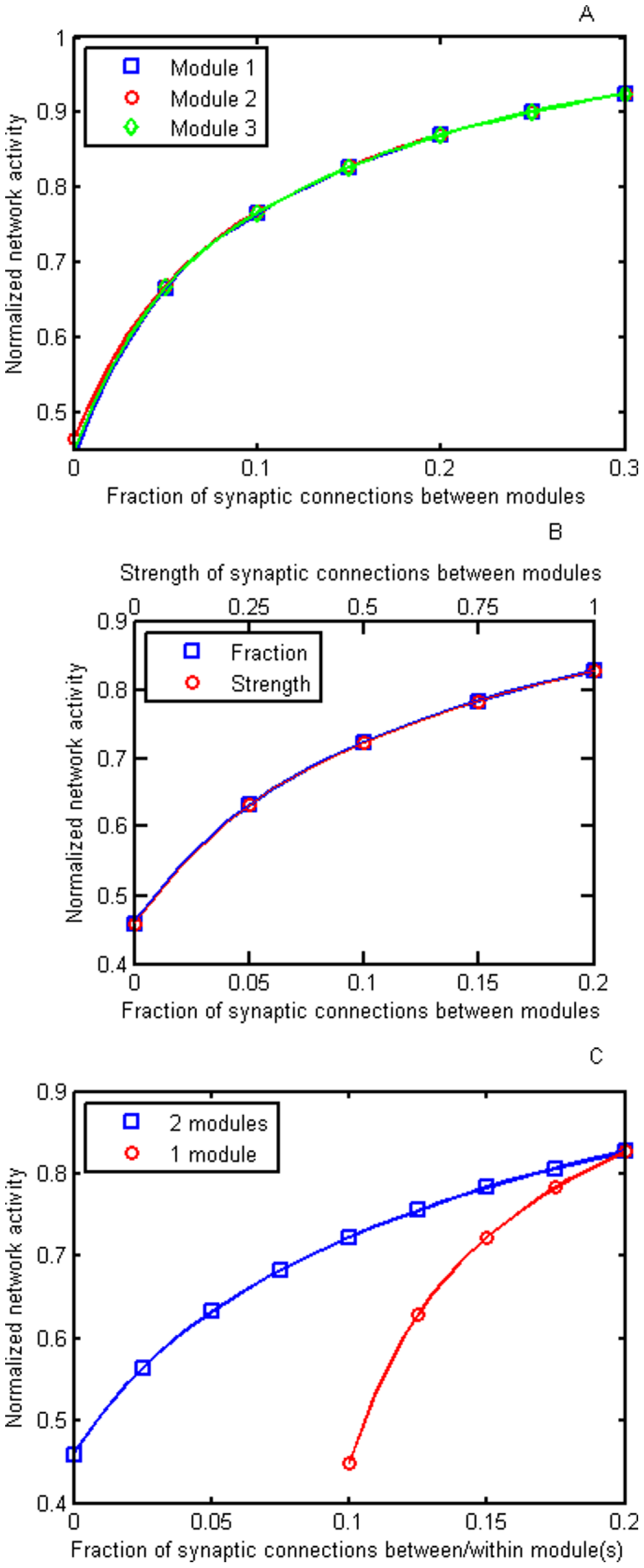
Results for the simulations of Model 2 giving changes in the activity in the modules (normalized by the maximum activity) with changes in inter-modular synaptic connections. A, comparison between activity and decreases in the fraction of inter-modular synaptic connections (*F_ext_*) for three modules (results for each of the three modules indicated by open squares, open circles and open triangles, respectively). Note that there is very little difference in the changes in the normalized activity with changes in the inter-modular extent of synaptic connections for any of the modules. The intra-modular synaptic connectivity fraction *F* was kept at 0.30 for each module and the coupling strength is *S* = 0.00075. The number of neurons in each module was 3267 (99×33). B, comparison between normalized activity in the modules and decreases in the fraction of inter-modular synaptic connections (*F_ext_*) between them on the one hand (open squares) and activity in the modules and decreases in the strength of synaptic connections on the other (open circles). This is a two modular case, with the results for just one module shown as the other one had identical activity; similar results were obtained for the three module case. The intra-modular synaptic connectivity *F* was kept at 0.2 and the coupling strength is *S* = 0.0015. The number of neurons in each module was 2450 (70×35). Note that for the values given in the abscissa there is no difference between changing the extent of inter-modular synaptic connections and changing the strength of the connections. C, comparison between normalized activity in the modules and decreases in the fraction of inter-modular synaptic connections (*F_ext_*) between them for the case when the intra-modular extent of synaptic connections was kept at 0.20 (open squares) and the case in which the extent of both inter-modular and intra-modular synaptic connectivity was reduced to the same extent (open circles); *S* = 0.0015 The number of neurons in each module was 2450 (70×35).

The extent of modular activity as measured using network activity with changes in the extent of synaptic coupling between modules (Figure 1) has been determined in simulations. These show that there is very little change in the curves relating network activity to the extent of synaptic coupling between modules (Figure 3A) for the three module case given in Figure 1. In the example shown for illustration, the intra-modular synaptic coupling fraction is fixed at *F* = 0.3.and initially the inter-modular coupling fraction *F_ext_* is given the same value; that is each neuron is on average connected to 0.3 of all the intra- and inter-modular neurons. Decreasing the number of inter-modular synaptic couplings by 24% decreases network activity by about 7%, and decreasing these couplings to zero decreases network activity by about 50% (Figure 3A). These values are similar to those for the computational solution (Figure 2) and so are similar to the observations in schizophrenia as noted above and indicated in detail below. Additional computations were performed for different sized networks and connection fractions. It was found that provided the initial connection fractions were the same (*F_ext_ = F*) then the decrease in network activity as *F_ext_* is reduced is similar to that shown in Figure 3A. (Some additional results are given in Figure S2.).

It should be noted that there is an equivalence between the extent of decrease in modular activity and the fraction of inter-modular synaptic connections on the one hand with the decrease in modular activity and the strength of the inter-modular synaptic connections on the other (Figure 3B). So either a loss of inter-modular synaptic connections or a decrease in the efficacy of synapses subserving these connections or a combination of both will retain the relation between modular activity and modular connectiveness.

Simulations allow a comparison to be made between the extent of decrease in network activity following a decrease in the inter-modular synaptic connections on the one hand and the decrease in network activity following a decrease in both inter-modular and intra-modular synaptic connections on the other. It is only necessary to decrease all the synaptic connections by half in order, in the example shown in Figure 3C, to halve network activity whereas, as previously shown (Figure 3A), if only inter-modular synaptic connections change then these must be reduced to zero in order to reduce network activity by 50%.

### Experimental: Observed Changes in the Functioning of Cortical Areas on Changing the Extent of their Inputs

#### CMRglc(ox) required to sustain local cortical networks in isolated cortical areas

**Figure 4:**
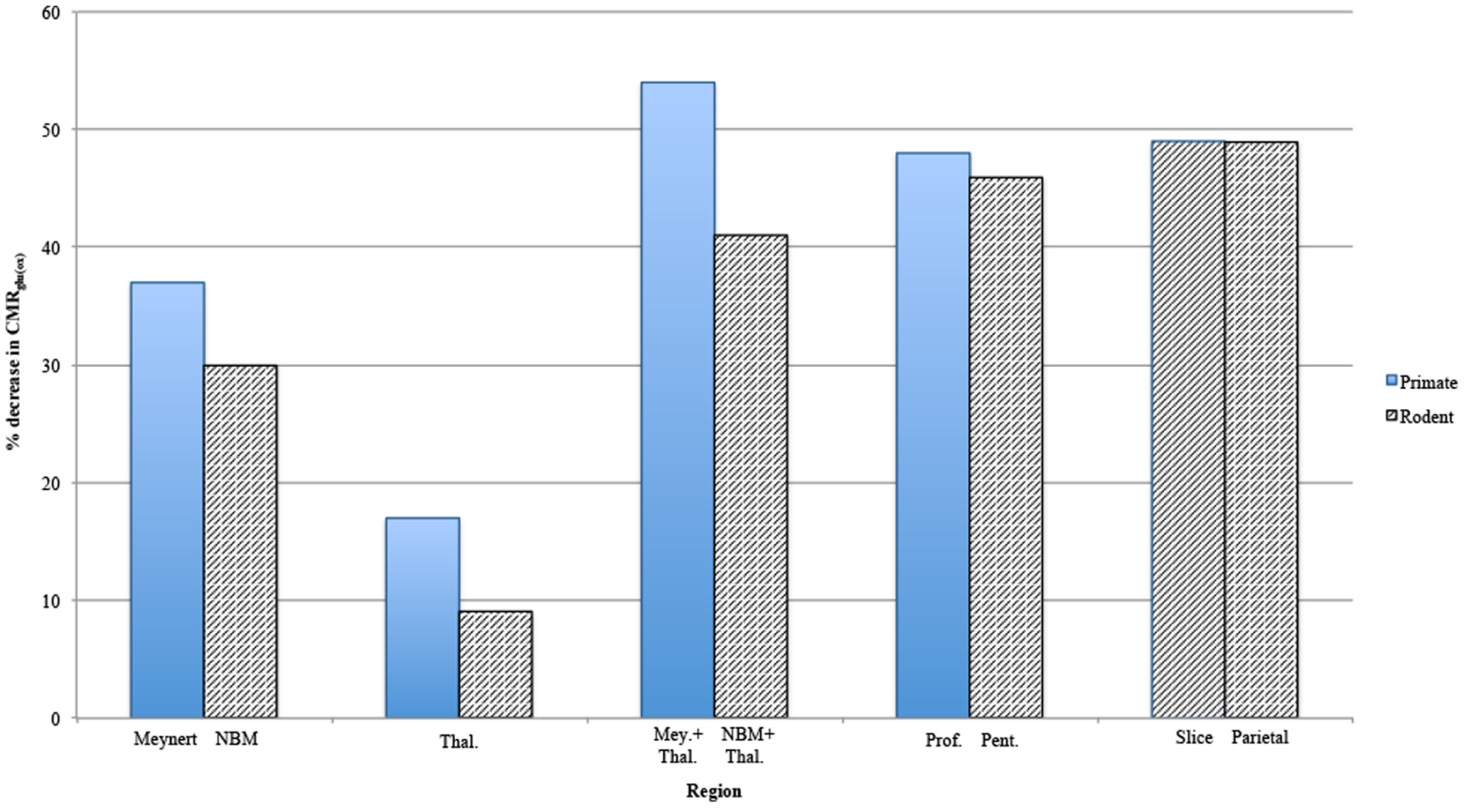
Histogram of the average percentage decreases in CMR_glc(ox)_ determined in rat and primate brains under the experimental conditions given in the abscissa. CMR_glc(ox)_ is proportional to network activity (Hyder *et al.,*2013). For primate (blue bars) these are from left to right: lesion of the nucleus of Meynert; the thalamic intralaminar nuclei (Thal); the nucleus of Meynert plus the thalamic nuclei (Mey+Thal); and under the anaesthetic propofol (Prof). For the rat (grey bars) these are from left to right: lesion of the nucleus basilis magnocellularis (NBM); the thalamic intralaminar nuclei (Thal); the NBM plus the thalamic nuclei (NBM+Thal); under the anaesthetic pentobarbital (pent); slices of cortex (slice); and lesion of the parietal cortex (Parietal). The percentages given in this histogram are from Tables S2 to S5.

Isolated slices of cortex possesses a CMR_glc(ox)_ in rats of 0.51±0.05 µmol/g/min, compared with the normal CMR_glc(ox)_of 1.01±0.03 µmol/g/min (Table S1), so there is a 49% decrease in CMR_glc(ox)_ (Figure 4). Thus the ongoing activity of the cortex remains high even in the absence of thalamic, forebrain and associational fiber connections. This decrease in CMR_glc(ox)_ of about 50% when the cortical area under consideration is deprived of its inputs is about the same as that predicted by both the computational (Figure 2) and simulation models (Figure 3) when the inter-modular synaptic connections are reduced to zero, given that CMR_glc(ox)_ is proportional to network electrical activity [31].

#### CMRglc(ox) required to sustain local cortical networks deprived of their long associational-fiber connections

Another test of the model is to consider the CMR_glc(ox)_ decrease in a local cortical area consequent on reducing most of the inputs into the area by removing major associational-fiber inputs. A cold lesion applied to the surface of the rat parietal cortex transiently blocks all activity in the underlying white matter including longitudinal association fibers [40]. The longitudinal fibers from parietal cortex are the most extensive in the brain. Medial and dorsal parietal lobe projects through superior longitudinal fasciculus components I, II and III as well as the fronto-occipital fasciculus and middle longitudinal fasciculus [5]. Lesion of these will disturb CMR_glc(ox)_ in the frontal and prefrontal, premotor/supplementary motor area and occipital cortex [5]. This is reflected in the measured CMR_glc(ox)_ changes following cold lesion of the parietal cortex, with decreases of 47±5% in frontal cortex (Table S2; from 1.0±0.03 to 0.53±0.05 µmol/g/min; [40], [41]), 51±8% in sensorimotor cortex (Table S2; from 1.07±0.04 to 0.52±0.05 µmol/g/min; [40], [41]) and in one measurement, 55% in the occipital cortex (Table S2; from 0.94±0.03 to 0.42±0.03 µmol/g/min; [40]–[42]). The average decrease in CMR_glc(ox)_ across the cortex is 49.2±2.5% (Table S2). Given that the parietal white matter lesions lead to the loss of a substantial part of the input to the named cortical areas then the 49% decrease in CMR_glc(ox)_ in these areas is in accord with the computational and simulation modeling (Figures 2 and 3).

#### CMRglc(ox) expenditure in cortical areas deprived of their brain-stem basal forebrain/intralaminar thalamic inputs

The excitability of a particular cortical area is also determined by the brain-stem basal forebrain and the intra-laminar thalamic inputs, besides the longitudinal associational inputs already considered. Although the cold lesions to the parietal cortex considered above may not substantially interfere with inputs from the basal forebrain and thalamus to cortical areas other than parietal, it is of considerable interest to determine this forebrain and thalamic contribution to cortical CMR_glc(ox)_.

Lesions to the nucleus basilis magnocellularis (NBM) in rodents, the homologue of Meynert nucleus in primates, gives 29±1% decrease in CMR_glc(ox)_ (Table S2 and Figure. 4; [43]). A lesion to the Meynert nucleus in primates reduces the normal CMR_glc(ox)_ by 37±9% in prefrontal, parietal, occipital and temporal cortex [44], [45]; (Table S4). There is then very substantial loss of CMR_glc(ox )_upon lesioning these nuclei.

CMR_glc(ox)_ measurements following lesioning of the intra-laminar thalamic input to the primate and rodent cortex from the centromedian, parafascicular, central lateral and midline nuclei have been made [46]. Lesioning of the thalamus gives rise to a widespread decrease of CMR_glc(ox)_ throughout the cortex, which is less than one third of that following lesioning of NBM or the nucleus of Meynert (see Tables S2(rat) and S4 (human); [47], [48]). These quantitative estimates of CMR_glc(ox)_ indicate that the basal forebrain nuclei play the dominant role. Simply adding forebrain and thalamic contributions to the CMR_glc(ox)_ suggests that about half of all cortical excitability, 54% in primates and 41% in rats, is dependent on these inputs (Figure 4).

The result of the calculation and modeling is that transient cold block lesioning of the parietal cortex, interrupting the main longitudinal associational projections between this parietal cortex and a number of other areas, gives rise to very substantial decreases in CMR_glc(ox)_, of about 50%, within frontal, occipital and sensorimotor area. It might be argued that the decrease of about 50% within frontal, occipital and sensorimotor areas on cold lesioning of the parietal cortex is not only due to interrupting the main long associational projections but is also due to interruption of the basal forebrain projections to these regions However for this to occur there would have to be very substantial retrograde loss of their projections for there is a rostro-caudal innervation of the cortex by the basal forebrain nuclei, with the frontal regions innervated well before the parietal [49]. Furthermore, the innervation of the hippocampus by the basal forebrain nuclei in the rat occurs well caudal to the basal forebrain nuclei [50], yet no significant changes in CMR_glc(ox)_ have been reported for the hippocampus following parietal lesions. It is suggested here that interruption of the parietal longitudinal associational fibers, the SLF, is primarily responsible for the loss of about 50% CMR_glc(ox)_ in the frontal regions, as well as in the sensorimotor area.

#### CMRglc(ox) expenditure required to sustain intrinsically determined as well as extrinsically determined excitability of local cortical networks

It is evident from Figure 4 that addition of the different sources of cortical excitability, measured using CMR_glc(ox)_, whether intrinsic, dependent on longitudinal associational fibers or on brain-stem basal forebrain/intralaminar thalamic inputs, adds up to about 150% of the normal awake CMR_glc(ox)_. Clearly one or more of these sources of excitability must be dependent on the other. Thus removal of the diffuse cholinergic innervation of the cortex by the brain stem nuclei leads to a 30% to 37% decrease of CMR_glc(ox)_ in rats and primates (Figure 4). If this is added to the decrease on lesioning the thalamus of 9% to 17% for rats and primates (Figure 4) then about half of the CMR_glc(ox)_ utilization in the cortex is dependent on these activating nuclei (Figure 4). This agrees with the known changes in CMR_glc(ox)_ during slow wave sleep in humans (38%, Table S3; [51] when the brain-stem excitability systems that project to the basal-forebrain nuclei and the non-specific nuclei of the thalamus are largely inactive. If this is the case then it might be argued that the diffuse cholinergic nuclei are necessary for the functioning of the networks composed by these associational fibers, with the major energy expenditure occurring as a consequence of the activity in the network not in energy expenditure required to sustain the direct input from the diffuse cholinergic nuclei. According to this scenario, these nuclei then trigger the activity in the associational-fiber networks, and this triggering does not consume much energy (Figure 1). If this is the case then about 50% of CMR_glc(ox)_ is expended in maintaining the local networks and 50% CMR_glc(ox)_ to maintaining the associational networks.

### Schizophrenia: Predicted and Observed Changes in the Functioning of Cortical Areas and their Inputs

Here we compare the changes in CMR_glc(ox)_ in the cortex of schizophrenia patients and the predictions of the model (Figure 1) following DTI determination of deterioration of their SLF.

#### The superior longitudinal fasciculus in schizophrenia: white matter deterioration measured with DTI

Fractional anisotropy decreases of 24.6±4.1% are measured in the SLF in schizophrenia (Table S5).

#### Cortical function in schizophrenia: decreases in CMRglc(ox) in cortical areas

Fourteen studies have noted changes in CMR_glc(ox)_ in different cortical areas of schizophrenia patients and seven studies measuring changes in regional cerebral blood flow (rCBF) in such patients (Table S6). The observations indicate percentage decreases in the cortex of 6% to 5% in frontal regions, 7% in temporal regions, and a lesser amount of 4% in parietal regions (Table S6). The predicted decrease from the model of Figure 1, as the graph in Figure 3B shows, is 6% for a 25% decrease in connectivity as measured with DTI. There is then good agreement between the predicted and observed decreases in cortical function following deterioration of the longitudinal fasciculus.

It will be noted in Table S6 that there is a 7% decrease in CMR_glc(ox)_ in the thalamus. As lesioning the intralaminar nuclei of the thalamus only gives rise to a 17% decrease in CMR_glc(ox)_ in the primate cortex (Figure 4), a 7% decrease in thalamic function is unlikely to contribute significantly to the decrease in cortical CMR_glc(ox)_. There are no measurements of CMR_glc(ox)_ in the basal nucleus of Meynert available in patients with schizophrenia; however recent observations suggest that the cholinergic pathways that mediate the projection of the nucleus of Meynert to the cortex are little compromised in schizophrenia [52].

Another consideration should be mentioned here and that is whether changes in intracortical synaptic connections are likely in schizophrenia, independent of changes in synaptic connections subserving the principal inter-cortical connections, like the superior longitudinal fasciculus. According to Figure 3C if both intercortical and intracortical connections are lost in schizophrenia there will be a much greater decrease in CMR_glc(ox)_ than when just the former are lost. Using fMRI and graph theory it has been claimed that there is a loss of intracortical connections in schizophrenia. If such a loss produces significant decreases over and above that due to the loss of intercortical connections then the present predictions will have to be modified.

## Discussion

### Modeling the Cortex in the Context of Schizophrenia: Cortico-cortical Synaptic Connections

The principal evidence in favor of the present model of the role of associational fibers in cortical function (Figure 1) is that changes in CMR_glc(ox)_ on removing associational connections, either by transient cold blocks or by lesions as in slices, is that predicted, given that electrical network activity is proportional to CMR_glc(ox)_ [31] (see Figures 3 and 4). However there are two caveats concerning the interpretation of the changes in the CMR_glc(ox)_ throughout the cortex following cold block of the rat parietal cortex (Table S2). One of these is that the distribution of fiber pathways has been determined for primate brain, using both autoradiography and DTI, to a high degree of accuracy [53], [54], whereas those for the rat brain have only recently become available [55], [56]. These show that there are reasonable homologies with respect to these pathways between the species: see for example the projection of the medial longitudinal fasciculus [57]; the cingulum bundle [58]; and the arcuate fasciculus [59], [60]. The identification of the fiber pathways interrupted in the experiments using transient cold block placed over the rat parietal cortex is to some extent dependent on these homologies so that the CMR_glc(ox)_ results in Table S2 must be considered with this reservation. Another reservation concerning Table S2 is that the values of CMR_glc(ox)_ used to determine the percentage decreases are the maximum observed and not those during the substantial recovery that often occurs over time in CMR_glc(ox)_.

Some mention should be made of the sources of experimental data, shown in the Tables (Supplement) and mostly summarized in Figure 4, which were used to test the models. The CMR_glc(ox)_ data presented in Tables S1, S2, S3, S4, S5 and S6 are all from in vivo experiments, with only that for rodent cortical slices in Table S1 being of necessity from in vitro experiments. Thus all the histogram bars in Figure 4, with the exception of the second last one (reading from left to right) are from in vitro measurements. The two species considered are rats and baboons and these are presented for comparison, as the changes in CMR_glc(ox)_ in the different experimental conditions are qualitatively very similar for both species (see Figure 4). The one comparison between in vivo and in vitro conditions is for the cortical slice versus the intact isolated parietal cortex (last two histogram bars in Figure 4) and it is of interest that these give the same changes in CMR_glc(ox)_.

Diffusion tensor imaging has noted decreases in fractional anisotropy changes in the uncinate fasciculus, joining the temporal lobe adjacent to the amygdala and the ventrolateral prefrontal cortex/orbitofrontal cortex, associated with emotional experiences, in schizophrenia [61], in addition to the changes noted above (Table S5) in the SLF (for a review of these pathways, see [19]). The measure of diffusivity in this imaging can be determined in three directions through a fiber tract, lambda I associated with the integrity of axons and lambdas II and III with that of myelin [62]–[65]. Changes in lambda II and lambda I predict the evolution in multiple sclerosis of severe tissue damage, including axonal swelling and loss of axons in a tract [66]. The full power of this approach has not yet been used on the principal fasciculi of the cortex in schizophrenia. It is interesting to note that changes in anisotropy of fasciculi in cognitive impairment is associated with a loss of gray matter in the cortical areas these fasciculi join [67].

### Modeling the Cortex in the Context of Schizophrenia: Subcortical-cortical Synaptic Connections

CMR_glc(ox)_almost doubles in the awake state from deep sleep, and changes in CMR_glc(ox)_ on removing associational connections in the awake animal are about those found in slices. This suggests that the activating system, comprising thalamic and basal nuclei, operates without much expenditure of energy to trigger functioning of the associational connections that are responsible for the differences between the awake and deep-sleep states. The model considered here for probing the role of associational fibers in cortical function (Figure 1) has been primarily tested by considering changes in CMR_glc(ox)_ following lesions to the cortex that interrupt these fibers. However, is the model consistent with changes in CMR_glc(ox)_ observed during sleep? This is now considered.

The primary form of brain damage in closed head injuries (Xu *et al.,* 2007) leading to a posttraumatic persistent vegetative state appear to be focal axonal injuries in the dorsolateral brain stem, including the ascending reticular activation [68]. Damage to the brain stem tegmentum, bilaterally, either in the pons alone or in the upper pons and midbrain, leads to coma whereas damage to the brain stem outside the tegmentum does not [69], [70]. Reversible inactivation of the brain-stem nuclei leads to an anesthesia-like state or coma [71]. It is therefore clear that the integrity of brain-stem structures is essential for maintaining consciousness. The mesopontine tegmental area of the brain stem projects to (i) intralaminar thalamic nuclei that in turn project over the cortex; (ii) to several pontomesencephalic, diencephalic and basal forebrain nuclei that also project to cortex, as well as to areas in the (iii) subcortical forebrain such as the hypothalamus, the septal area, the zona incerta and striato-pallidal system with some (iv) direct projections to the frontal cortex [72]. The ascending brain stem arousal system modulates the thalamo-cortical system, principally via the intralaminar thalamic nuclei, and has often been considered to be a major source of neocortical electrical activity as measured by the electroencephalogram during cortical arousal in wakefulness [73]–[75]. The ascending brain stem arousal system projection to basal forebrain neurons gives rise to activity in the neurons that correlates with wake-sleep patterns and electroencephalographic waveforms [2], [76], with some suggesting that these basal nuclei play a more diffuse role in cortical arousal than that provided by the thalamic nuclei [77]. However lesions or local inactivation of the thalamic nuclei with lidocaine does not antagonize persistent cortical activation [78]–[81]. On the other hand injections of the local anesthetic procaine into the basal forebrain does [82] as do lesions of this nucleus [83], [84]. Recently it has been shown that extensive thalamic lesions have little effect on electroencephalographic or behavoural measures of wakefulness whereas animals with large basal forebrain lesions or lesions to the brain stem parabrachial-precoeruleus are behaviourally unresponsive and possess a monotonous sub-1 Hz electroencephalogram during continuous gentle handling [85]. Lesions to the nucleus basalis of Meynert (baboon) or the nucleus basilis magnocellularis (rat) removes substantial cholinergic innervation of the principal lobes of the cortex, including prefrontal, parietal, temporal, occipital and cingulate cortex [49]. Lesions to the remaining basal forebrain nuclei (in the rat; the diagonal band of Broca and the medial septal nuclei) removes cholinergic innervation from the hippocampus (including subiculum and entorhinal cortex) and medial mesolimbic cortex and part of the occipital cortex [50], so that it appears that these nuclei play a principal role in maintaining cortical excitability at a level sufficient for consciousness.

Alterations between being awake and sleep are determined by groups of neurons in the ascending reticular activating systems, which activate cholinergic neurons in the basal forebrain that fire during waking but markedly decrease their firing during slow-wave sleep in contrast to GABAergic neurons (principally in the basal forebrain that act in the opposite way [86]. In the prefrontal cortex layer 3 pyramidal neurons are the principal origins of cortico-cortical connections, including those of the SLF [87]. The primary effect of the acetylcholine released from the NBMin rodents and the nucleus of Meynert in primates is to act on m_2_ muscarinic receptors found on synaptic spines, where they facilitate synaptic transmission in the long projection fiber tracts (for a recent summary of these facilitatory mechanisms, see [88]. The projection of these nuclei to the cortex may be regarded as serving the role of supplying a tuneable effect that controls the efficacy of inter-modular connections. This amounts to changing the parameters *g_ext_* (Model 1) or *S_ext_* and/or *F_ext_* (Model 2).In visual cortex at least 50% of neurons in layer 2/3 possess these muscarinic receptors (Figure 2 in [88], which overwhelmingly produces a facilitating effect on glutamatergic transmission to these neurons [88]; see their Figure. 8B) The m_1_ and m_2_ receptors are on spines that are contacted by noncholinergic afferents [89]–[92] so that transmission from cholinergic afferents is very likely to involve the diffusion of acetylcholine from the terminals over relatively long distances as in volume transmission, as described by Fuxe and colleagues [93] and as occurs prominently in the peripheral autonomic nervous system [94]. Such transmission involves activation of G-proteins that give rise to effects that typically operate in the seconds time domain [95]. Of particular interest is that these facilitatory effects due to acetylcholine are maintained for over an hour after cessation of the action of acetylcholine on muscarinic receptors [96], [97], perhaps indicating very little energy expenditure to maintain the facilitatory state after the acetylcholine has ceased to be released.

Transcranial magnetic stimulation (TMS) of the cortex during slow wave sleep reveals, as in the case of anaesthetics, that activation only occurs at the site/area stimulated [98], with the subject declaring no recollection of any consciousness during the period [99]. However in rapid eye movement sleep, when subjects are unresponsive to sensory stimuli but rather have dreams which they report on when awake, the cortical response to TMS again shows sequential activation throughout the cortex [100], as in the awake state. These observations suggest that the longitudinal associational connections are not active in slow-wave sleep as they are when awake, and confirm the present hypothesis concerning the functioning of the brain-stem basal forebrain/intralaminar thalamic cortical network.

### Implications of the Cortical Model in Schizophrenia

The model that emerges from this work is that about 50% of CMR_glc(ox)_ is required to sustain spontaneous activity of different cohorts of neurons in local networks representing different aspects of a cortical areas functions, such as orientation in V1. Input from the thalamus triggers sets of these cohorts through a relatively low CMR_glc(ox)_ consumption mechanism. The remaining 50% of CMR_glc(ox)_ is primarily required to sustain spontaneous activity in the associational networks representing global aspects of the functions of cortical areas, such as those involving V1 and MST in the visual system. The triggering of these associational networks by basal forebrain nuclei and from the intralaminar nuclei of the thalamus also consumes very little CMR_glc(ox)_ consumption.

In summary, a cortical model has been developed that quantitatively conforms with levels of electrical activity, measured using CMR_glc(ox),_ under different experimental conditions. These include the extent of changes in activity in cortex when isolated from all longitudinal and subcortical synaptic connections, when just longitudinal synaptic connections are removed and when subcortical synaptic connections are removed. The model quantitatively predicts the extent of decrease in activity observed in the cortex following the observed changes in function of longitudinal tract integrity but predicts a much greater change in cortical activity if significant changes in synaptic connections occur throughout the cortex (Figure 3C). This work points to the longitudinal associational fascicules as a major area of investigation in schizophrenia, a point emphasized in a recent review [101], for their deterioration explains both the observed changes in gray matter activity as well as the loss of synapses in the gray matter due to loss of associational synaptic connections.

## Supporting Information

Figure S1 **Spike trains for three neurons in a Model 2 network.** In the first 500 ms the neurons are being driven by a random Poisson input which is then removed and the neurons settle into a stable firing pattern.
(TIF)

Figure S2 **Results for simulations of Model 2 for the case of two identical modules, showing changes in the network activity in one module with changes in inter-modular synaptic connections.** A. Decreases in network activity as the fraction of inter-modular connections (*F_ext_*) is reduced, for 3 values of the intra-modular connection fraction, *F.* Each module contains 2450 neurons; the value *S = *0.0015 is appropriate for this size of network and for the connection fraction *F* = 0.2. It is seen that starting with an inter-modular connection fraction *F_ext_* = *F* = 0.2 (blue squares) gives the desired decrease, whereas starting with *F_ext_* = 0.25 (red circles) or *F_ext_* = 0.15 (green diamonds) gives decreases that are either too small or too large. B. Decreases in network activity as the fraction of inter-modular connections (*F_ext_*) is reduced, for 4 different sized networks; *N* is the total number of neurons and each module contains half this number. The intra-modular connection fraction is *F* = 0.2 for all cases, and the appropriate *S* values are shown for each size of network. It is seen that the results converge as the network size increases.
(TIF)

Table S1 Experimental results for average CMR_glc(ox)_ determinations (µmol/g/min) for the cortex of rodents in the conditions indicated [41]–[43], [102]–[123].
(DOCX)

Table S2 Rodent changes in CMR_glc(ox)_ following lesions. Changes in CMR_glc(ox)_ in (µmol/g/min) in the cortical regions listed in the first column following lesions to the structures named in the first row. In each case the upper CMR_glc(ox)_ is the control value and the lower CMR_glc(ox)_ that following the lesion. The percentage change in each case is given in parenthesis [40], [42], [43], [47], [124]. **Key to first row**: NBM, nucleus basilis magnocellularis; Thalam, thalamus; Pariet, parietal cortex.
(DOCX)

Table S3 Experimental results for average CMR_glc(ox)_ determinations (µmol/g/min) for the cortex of humans in the conditions indicated [51], [125]–[138].
(DOCX)

Table S4 Primate changes in CMR_glc(ox)_ following lesions. Changes in CMR_glc(ox)_ (µmol/g/min) in the cortical regions listed in the first column following lesions to the structures named in the first row. In each case the upper CMR_glc(ox)_ is the control value and the lower CMR_glc(ox)_ that following the lesion. The percentage decrease in each case is given in parenthesis [44], [48]. **Key to first row**: Meyn, nucleus of Meynert; Rhinal, rhinal cortex; Thal, thalamus.
(DOCX)

Table S5 Fractional anisotropy decrease of the superior longitudinal fasciculus in schizophrenia [19], [139]–[142].
(DOCX)

Table S6 Percentage changes in CMG_glc(ox)_ (rows 1–14) and rCBF (rows 15–21) in schizophrenia [32], [114], [143]–[161]. Note: Two papers report increases rather than decreases in rCBF in prefrontal regions of schizophrenia patients ([162], [163]), with [162] reporting no differences in a subsequent paper [161]. These have not been included in this Table.
(DOCX)
