# Supplementary figures and images for: Fiber Pathway Pathology, Synapse Loss and Decline of Cortical Function in Schizophrenia
Source: PLoS One. 2013 Apr 8;8(4):e60518. doi: 10.1371/journal.pone.0060518 (PMC3620229; doi:10.1371/journal.pone.0060518)

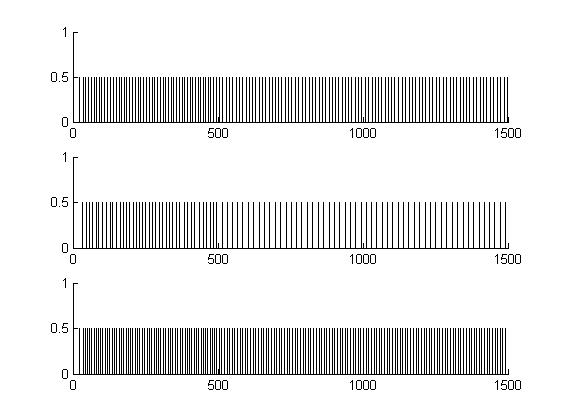

Supplement: Figure S1 — Spike trains for three neurons in a Model 2 network. In the first 500 ms the neurons are being driven by a random Poisson input which is then removed and the neurons settle into a stable firing pattern. (TIF) [file pone.0060518.s001.tif]

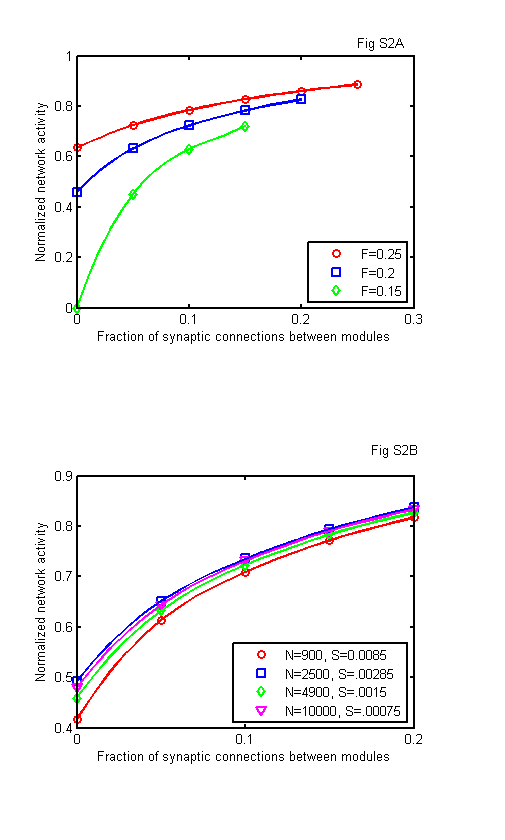

Supplement: Figure S2 — Results for simulations of Model 2 for the case of two identical modules, showing changes in the network activity in one module with changes in inter-modular synaptic connections. A. Decreases in network activity as the fraction of inter-modular connections (Fext) is reduced, for 3 values of the intra-modular connection fraction, F. Each module contains 2450 neurons; the value S = 0.0015 is appropriate for this size of network and for the connection fraction F = 0.2. It is seen that starting with an inter-modular connection fraction Fext = F = 0.2 (blue squares) gives the desired decrease, whereas starting with Fext = 0.25 (red circles) or Fext = 0.15 (green diamonds) gives decreases that are either too small or too large. B. Decreases in network activity as the fraction of inter-modular connections (Fext) is reduced, for 4 different sized networks; N is the total number of neurons and each module contains half this number. The intra-modular connection fraction is F = 0.2 for all cases, and the appropriate S values are shown for each size of network. It is seen that the results converge as the network size increases. (TIF) [file pone.0060518.s002.tif]
