# Supplementary material for: Fiber Pathway Pathology, Synapse Loss and Decline of Cortical Function in Schizophrenia
Source: PLoS One. 2013 Apr 8;8(4):e60518. doi: 10.1371/journal.pone.0060518 (PMC3620229; doi:10.1371/journal.pone.0060518)
Supplement: Table S1 — Experimental results for average CMRglc(ox) determinations (µmol/g/min) for the cortex of rodents in the conditions indicated [41]–[43], [102]–[123]. (DOCX) [file pone.0060518.s003.docx]

**Table S1**

| Reference | Condition | CMR_glc(ox)_ |
| --- | --- | --- |
| [[102](#_ENREF_102)] | Rat, awake | 0.92 |
| [[43](#_ENREF_43)] | Rat, awake | 0.89 |
| [[42](#_ENREF_42)] | Rat, awake | 1.10 |
| [[41](#_ENREF_41)] | Rat, awake | 1.09 |
| [[103](#_ENREF_103)] | Rat, awake | 1.12 |
| [[104](#_ENREF_104)] | Rat, awake | 1.09 |
| [[105](#_ENREF_105)] | Rat, awake | 1.07 |
| [[106](#_ENREF_106)] | Rat, awake | 0.73 |
| [[107](#_ENREF_107)] | Rat, awake | 1.03 |
| [[108](#_ENREF_108)] | Rat, awake | 1.11 |
| [[109](#_ENREF_109)] | Rat, awake | 1.00 |
| [[110](#_ENREF_110)] | Rat, awake | 0.96 |
| [[111](#_ENREF_111)] | Rat, cortex slice | 0.35 |
| [[112](#_ENREF_112)] | Rat, cortex slice | 0.42 |
| [[113](#_ENREF_113)] | Rat, cortex slice | 0.60 |
| [[114](#_ENREF_114)] | Rat, motor cortex slice | 0.77 |
| [[115](#_ENREF_115)] | Rat, hippocampus slice | 0.37 |
| [[116](#_ENREF_116)] | Rat, hippocampus slice | 0.56 |
| [[117](#_ENREF_117)] | Rat, hypothalamic slice | 0.65 |
| [[118](#_ENREF_118)] | Rat, hypothalamic slice | 0.58 |
| [[119](#_ENREF_119)] | Rat, suprachiasmatic slice | 0.50 |
| [[120](#_ENREF_120)] | Rat, suprachiasmatic slice | 0.38 |
| [[121](#_ENREF_121)] | Guinea-pig suprachiasmatic slice | 0.78 |
| [[122](#_ENREF_122)] | Guinea-pig cortex slice | 0.70 |
| [[123](#_ENREF_123)] | Rat, pentobarbital (50 mg/kg) | 0.40 |
| [[105](#_ENREF_105)] | Rat, pentobarbital (50mg/kg) | 0.65 |
| [[110](#_ENREF_110)] | Rat, pentobarbital (50mg/kg) | 0.57 |
| [[106](#_ENREF_106)] | Rat, ketamine/zylazine | 0.42 |
| [[108](#_ENREF_108)] | Rat, ketamine/diazepam | 0.82 |
| [[109](#_ENREF_109)] | Rat, ketamine (30 mg/kg) | 0.85 |
| [[107](#_ENREF_107)] | Rat, chlorolase | 0.37 |
| [[103](#_ENREF_103)] | Rat, thiopental | 0.65 |
