# Supplementary material for: Fiber Pathway Pathology, Synapse Loss and Decline of Cortical Function in Schizophrenia
Source: PLoS One. 2013 Apr 8;8(4):e60518. doi: 10.1371/journal.pone.0060518 (PMC3620229; doi:10.1371/journal.pone.0060518)
Supplement: Table S2 — Rodent changes in CMRglc(ox) following lesions. Changes in CMRglc(ox) in (µmol/g/min) in the cortical regions listed in the first column following lesions to the structures named in the first row. In each case the upper CMRglc(ox) is the control value and the lower CMRglc(ox) that following the lesion. The percentage change in each case is given in parenthesis [40], [42], [43], [47], [124]. Key to first row: NBM, nucleus basilis magnocellularis; Thalam, thalamus; Pariet, parietal cortex. (DOCX) [file pone.0060518.s004.docx]

**Table S2**

|  | NBM | Thalam | Pariet | Pariet | Pariet |
| --- | --- | --- | --- | --- | --- |
| References | [[43](#_ENREF_43)] | [[47](#_ENREF_47)] | [[124](#_ENREF_124)] | [[42](#_ENREF_42)] | [[40](#_ENREF_40)] |
| Dorsolateral  prefrontal | 0.76±0.10  0.55±0.9  (28) | 0.93±0.09  0.83±0.11  (11) | 1.07±0.29  0.56±0.14  (48) | 0.94±0.03  0.44±0.03  (53) | 1.01±0.07  0.60±0.04  (41) |
| Orbitolateral  prefrontal | 0.89±0.13  0.60±0.17  (33) | 1.49±0.08  1.37±0.07  (8) |  |  |  |
| Sensorimotor | 0.83±0.09  0.57±0.13  (31) |  | 1.15±0.24  0.55±0.14  (52) | 1.02±0.04  0.42±0.06  (59) | 1.05±0.06  0.60±0.04  (43) |
| Parietal | 0.79±0.12  0.56±0.16  (29) | 1.07±0.09  0.96±0.18  (10) |  |  |  |
| Occipital | 0.76±0.09  0.57±0.11  (25) |  |  | 0.94±0.03  0.42±0.03  (55) |  |
| Average  % change | (29±1) | (9±2) | (49±3) | (55±3) | (42±2) |
