# Supplementary material for: Fiber Pathway Pathology, Synapse Loss and Decline of Cortical Function in Schizophrenia
Source: PLoS One. 2013 Apr 8;8(4):e60518. doi: 10.1371/journal.pone.0060518 (PMC3620229; doi:10.1371/journal.pone.0060518)
Supplement: Table S3 — Experimental results for average CMRglc(ox) determinations (µmol/g/min) for the cortex of humans in the conditions indicated [51], [125]–[138]. (DOCX) [file pone.0060518.s005.docx]

**Table S3**

| Reference | Condition | CMR_glc(ox)_ |
| --- | --- | --- |
| [[125](#_ENREF_125)] | awake | 0.45 |
| [[51](#_ENREF_51)] | awake | 0.21 |
| [[126](#_ENREF_126)] | awake | 0.33 |
| [[127](#_ENREF_127)] | awake | 0.36 |
| [[128](#_ENREF_128)] | awake | 0.33 |
| [[129](#_ENREF_129)] | awake | 0.42 |
| [[130](#_ENREF_130)] | awake | 0.38 |
| [[131](#_ENREF_131)] | awake | 0.29 |
| [[132](#_ENREF_132)] | awake | 0.35 |
| [[133](#_ENREF_133)] | awake | 0.37 |
| [[134](#_ENREF_134)] | awake | 0.36 |
| [[135](#_ENREF_135)] | awake | 0.27 |
| [[51](#_ENREF_51)] | non-rapid eye movement sleep | 0.14 |
| [[136](#_ENREF_136)] | propofol | 0.16 |
| [[126](#_ENREF_126)] | propofol | 0.18 ? |
| [[137](#_ENREF_137)] | halothane | 0.19 |
| [[138](#_ENREF_138)] | sevoflurane | 0.19? |
