# Supplementary material for: Fiber Pathway Pathology, Synapse Loss and Decline of Cortical Function in Schizophrenia
Source: PLoS One. 2013 Apr 8;8(4):e60518. doi: 10.1371/journal.pone.0060518 (PMC3620229; doi:10.1371/journal.pone.0060518)
Supplement: Table S4 — Primate changes in CMRglc(ox) following lesions. Changes in CMRglc(ox) (µmol/g/min) in the cortical regions listed in the first column following lesions to the structures named in the first row. In each case the upper CMRglc(ox) is the control value and the lower CMRglc(ox) that following the lesion. The percentage decrease in each case is given in parenthesis [44], [48]. Key to first row: Meyn, nucleus of Meynert; Rhinal, rhinal cortex; Thal, thalamus. (DOCX) [file pone.0060518.s006.docx]

**Table S4.**

|  | Meyn | Thal |
| --- | --- | --- |
| References | [[44](#_ENREF_44)]; baboon | [[48](#_ENREF_48)]; human |
| Dorsolateral  prefrontal | 0.28±0.02  0.15±0.02  (46) | 0.40±0.04  0.32±0.02  (20) |
| Orbitolateral  prefrontal |  |  |
| Sensorimotor |  | 0.40±0.04  0.33±0.02  (18) |
| Parietal | 0.26±0.01  0.18±0.02  (30) | 0.38±0.04  0.33±0.04  (13) |
| Anterior  temporal | 0.30±0.02  0.17±0.02  (43) | 0.36±0.03  0.31±0.02  (14) |
| Primary  occipital | 0.24±0.01  0.17±0.02  (29) | 0.40±0.08  0.35±0.02  (13) |
| Anterior  cingulate |  | 0.44±0.03  0.35±0.04  (20) |
| Insula |  | 0.40±0.05  0.32±0.03  (20) |
| Average  % change | (37±9) | (17±3) |
