# Supplementary material for: Fiber Pathway Pathology, Synapse Loss and Decline of Cortical Function in Schizophrenia
Source: PLoS One. 2013 Apr 8;8(4):e60518. doi: 10.1371/journal.pone.0060518 (PMC3620229; doi:10.1371/journal.pone.0060518)
Supplement: Table S5 — Fractional anisotropy decrease of the superior longitudinal fasciculus in schizophrenia [19], [139]–[142]. (DOCX) [file pone.0060518.s007.docx]

**Table S5**

| Percentage change in fractional anisotropy (%) | Authors | Table or figure |
| --- | --- | --- |
| - 33 | [[19](#_ENREF_19)] | Fig. 2 |
| -24 | [[139](#_ENREF_139)] | Table 1 |
| -20 | [[140](#_ENREF_140)] | Fig. 3 |
| -12 | [[141](#_ENREF_141)] | Table 2 |
| -34 | [[142](#_ENREF_142)] | Table 1 |
| Average -24.6 ± 4.1 |  |  |
