# Supplementary material for: Fiber Pathway Pathology, Synapse Loss and Decline of Cortical Function in Schizophrenia
Source: PLoS One. 2013 Apr 8;8(4):e60518. doi: 10.1371/journal.pone.0060518 (PMC3620229; doi:10.1371/journal.pone.0060518)
Supplement: Table S6 — Percentage changes in CMGglc(ox) (rows 1–14) and rCBF (rows 15–21) in schizophrenia [32], [114], [143]–[161]. Note: Two papers report increases rather than decreases in rCBF in prefrontal regions of schizophrenia patients ([162], [163]), with [162] reporting no differences in a subsequent paper [161]. These have not been included in this Table. (DOCX) [file pone.0060518.s008.docx]

**Table S6**

| AuthorAutReferences | Frontal | DLPFC | Parietal | Temporal | Thalamus |
| --- | --- | --- | --- | --- | --- |
| [[143](#_ENREF_143)] | -4.0 |  |  |  |  |
| [[144](#_ENREF_144)] | -15.0 |  |  | -14.0 |  |
| [[145](#_ENREF_145)] | -5.0 |  | -2.0 |  |  |
| [[146](#_ENREF_146)] | -3.4 |  |  |  |  |
| [[147](#_ENREF_147)] | -5.0 |  |  |  |  |
| [[148](#_ENREF_148)] |  | -4.4 |  |  |  |
| [[149](#_ENREF_149)] | -12.0 |  | -13.0 | -13.0 |  |
| [[150](#_ENREF_150)] | -6.7 |  |  |  |  |
| [[151](#_ENREF_151)] | -4.8 |  | -2.7 | -2.8 | -4.5 |
| [[152](#_ENREF_152)] |  |  |  |  |  |
| [[153](#_ENREF_153)] | -4.5 |  | -1.0 | -1.2 | +1 |
| [[154](#_ENREF_154)] |  |  |  |  | -8.1 |
| [[155](#_ENREF_155)] |  |  |  |  | -9.5 |
| [[156](#_ENREF_156)] |  |  |  |  | -9.0 |
| [[157](#_ENREF_157)] |  | -4.9 | -4.7 | -6.0 | -10.9 |
| [[158](#_ENREF_158)] |  | -5.0 |  |  |  |
| [[159](#_ENREF_159)] | -5.0 |  | -0.5 | -3.5 |  |
| [[32](#_ENREF_32)] | -8.5 |  | -5.8 | -10.5 |  |
| [[160](#_ENREF_160)] | -3.1 |  |  |  |  |
| [[161](#_ENREF_161)] | -0.6 |  |  |  |  |
| [[114](#_ENREF_114)] |  | -4.2 |  |  |  |
| Average ± SEM | -6.0 ± 1.1 | -4.6 ±  0.2 | -4.2 ±  1.6 | -7.1 ±  1.9 | -6.8 ±  1.8 |
